# Extracellular matrix turnover and inflammation in chemically-induced TMJ arthritis mouse models

**DOI:** 10.1371/journal.pone.0223244

**Published:** 2019-10-11

**Authors:** Mallory Morel, Angela Ruscitto, Serhiy Pylawka, Gwendolyn Reeve, Mildred C. Embree

**Affiliations:** 1 TMJ Biology and Regenerative Medicine Laboratory, College of Dental Medicine, Columbia University Irving Medical Center, New York, NY, United States of America; 2 Division of Oral and Maxillofacial Surgery, New York Presbyterian Weill Cornell Medical Center, New York, NY, United States of America; University of Umeå, SWEDEN

## Abstract

The temporomandibular joint (TMJ) is a fibrocartilaginous tissue critical for chewing and speaking. In patients with temporomandibular disorders (TMDs), permanent tissue loss can occur. Recapitulating the complexity of TMDs in animal models is difficult, yet critical for the advent of new therapies. Synovial fluid from diseased human samples revealed elevated levels of tumor necrosis factor alpha (TNF-alpha). Here, we propose to recapitulate these findings in mice by subjecting murine TMJs with TNF-alpha or CFA (Complete Freund’s Adjuvant) in mandibular condyle explant cultures and by local delivery *in vivo* using TMJ intra-articular injections. Both TNF-alpha and CFA delivery to whole mandibular explants and *in vivo* increased extracellular matrix deposition and increased cartilage thickness, while TNF-alpha treated explants had increased expression of inflammatory cytokines and degradative enzymes. Moreover, the application of TNF-alpha or CFA in both models reduced cell number. CFA delivery *in vivo* caused soft tissue inflammation, including pannus formation. Our work provides two methods of chemically induced TMJ inflammatory arthritis through a condyle explant model and intra-articular injection model that replicate findings seen in synovial fluid of human patients, which can be used for further studies delineating the mechanisms underlying TMJ pathology.

## Introduction

The temporomandibular joint (TMJ) is critical for mastication, occlusion, and speech. Temporomandibular disorders (TMDs) affect over 10–15% of the adult population [1] and describe a group of complicated and diverse conditions that affect the TMJ and associated ligaments and musculature [2]. TMDs can result in regional pain and TMJ dysfunction, including limited joint movements, such as trismus and jaw interferences [3, 4]. TMDs can be further classified into three categories including myofascial pain disorders, articular disorders and degenerative disorders [2, 5, 6]. Myofascial pain is among the most common TMD diagnosis [7], but is not always associated with TMJ articular changes. Articular disorders involve abnormal disc position, including anterior disc displacement with and without reduction, also referred to as internal derangement [2]. Chronic articular disorders can lead to joint mechanical instability and secondary osteoarthritis (OA) and progressive degeneration [5, 8]. Interestingly, TMDs can also be associated with co-morbidities, such as referred pain, headaches, and irritable bowel syndrome [9, 10]. Moreover the etiology of TMDs is multifactorial and has been attributed to multiple genetic, environmental, behavioral and psychological factors [2, 9, 11–13].

The cartilaginous tissue of the TMJ is a complex tissue that consists of chondrocytes embedded in an abundant extracellular matrix (ECM). The makeup of the ECM is primarily collagens, proteoglycans, and water. In homeostasis, chondrocytes within the ECM maintain a balance between synthesis and degradation with a resulting low turnover of matrix proteins [14]. Disruption of this balance due to disease results in a biphasic response, with an early stage marked by changes in quantity and distribution of ECM in which the chondrocytes attempt to repair the damaged ECM. Late stage is marked by a gradual increase of the degradative process that results in a net loss of ECM [15]. These processes are influenced by cytokines and growth factors; primarily, both TNF-alpha and interleukin-1beta (IL-1beta) are implicated in the destruction of articular cartilage through regulation of matrix metalloproteinases (MMPs) that affect matrix synthesis [16]. The goal of therapy for TMDs would be to provide relief to patients suffering from this disease prior to the onset of the late stage of disease, degradative changes. Therefore, an exploration of the changes in the cartilage of the TMJ during the acute, early stage provides value to the study of inflammatory arthritis of the TMJ.

Given the complexity of TMDs clinical presentation and etiology, the development of animal models that precisely recapitulate the complexity of TMDs is unlikely. Currently, there are several types of TMJ osteoarthritis animal models, including genetically modified mice [8, 17], surgically induced TMJ injury models [18–21], and chemically induced inflammatory rat and rabbit models [19, 22]. Given the difficulty of TMJ surgery in rodent models, inducing TMJ inflammatory arthritis through genetic or chemical means is more feasible. The advantage of using a chemically induced rodent model is the ability to control the introduction of pathological agents. Here we subjected mouse TMJs to chemical mediators of arthritis using both whole mandible explants and *in vivo* using intra-articular injections. Taken together, we report a comprehensive analysis of chemically induced TMJ arthritis mouse models critical for understanding different TMJ cell populations involved in TMJ disease and for testing the efficacy of potential therapies.

## Results

### Cartilage degradation and high TNF-alpha levels in synovial fluid from patients with inflammatory TMJ pathology

**Fig 1:**
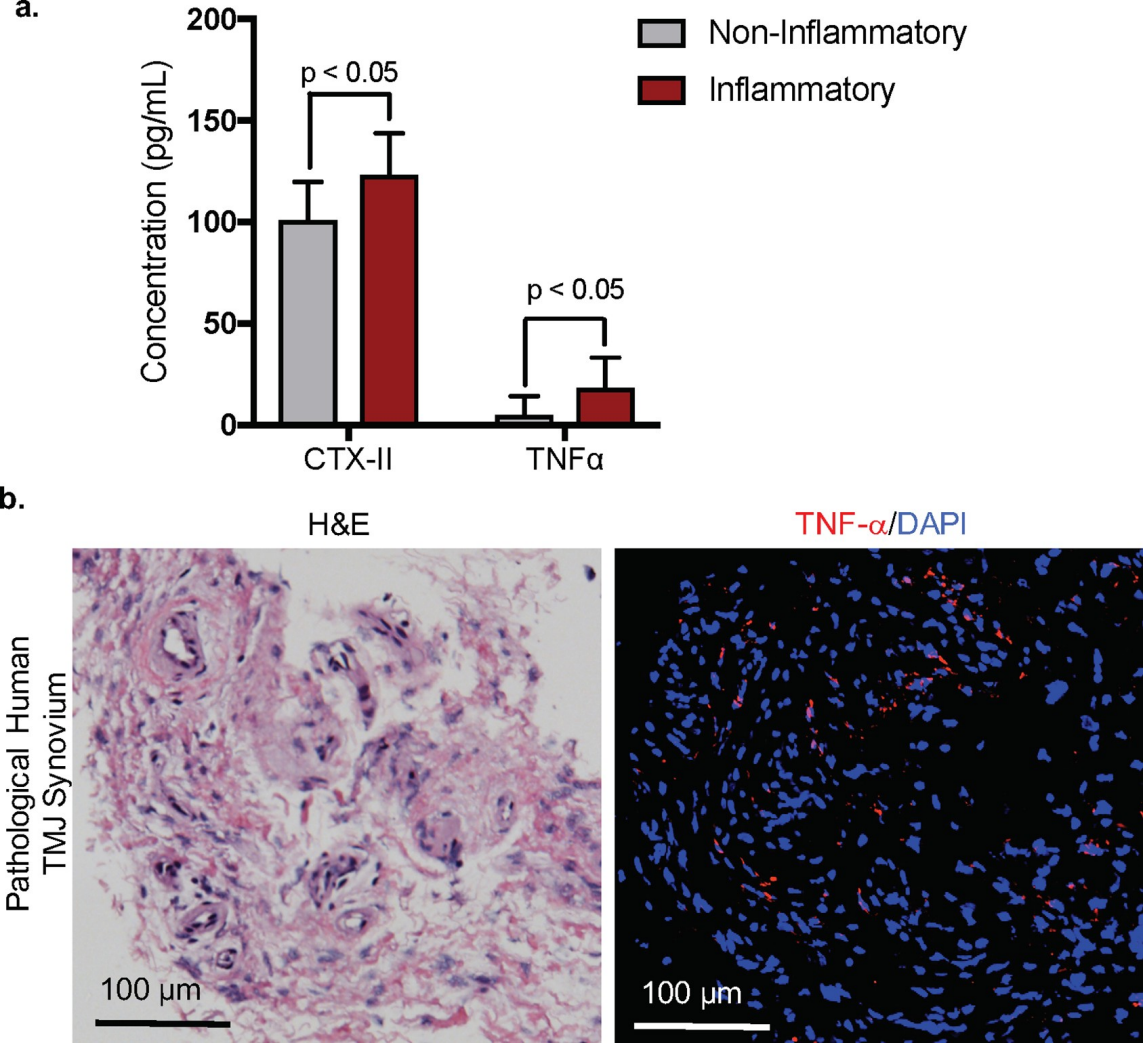
Synovial fluid and tissue derived from humans with inflammatory TMJ pathology have high CTXII and TNF-alpha levels. *(a)* ELISA was used to measure CTX-II, a type II collagen telopeptide as a biomarker for degradation, in synovial fluid derived from patients undergoing TMJ treatment. Patients with inflammatory TMJ pathology (n = 12; inclusion criteria included synovitis, osteoarthritis, and capsulitis) were included as ‘inflammatory’ and patients diagnosed with non-inflammatory TMJ pathology (n = 7; contusion, chronic dislocations, TMJ pain dysfunction syndrome, and disc displacement/internal derangement without associating synovitis) were labeled as ‘non-inflammatory.’ Data are mean ± SD; n = 2 technical replicates. * p<0.05, ordinary one-way ANOVA. *(b)* ELISA was used to measure TNF-alpha, an inflammatory mediator of joint arthritis, in synovial fluid derived from patients undergoing TMJ therapy. Patients identified with inflamed TMJ tissue (n = 12; inclusion criteria included synovitis, osteoarthritis, and capsulitis) were categorized as ‘inflammatory’ and patients with non-inflammatory TMJ pathology (n = 7; contusion, chronic dislocations, TMJ pain dysfunction syndrome, and disc displacement/internal derangement without associating synovitis) were categorized as ‘non-inflammatory.’ Data are mean ± SD; n = 2 replicates; *p<0.05, ordinary one-way ANOVA. *(c)* H&E tissue section of human TMJ synovial tissue from a patient diagnosed with TMJ synovitis (left). Immunohistochemistry confirmed that TNF-alpha (red) was expressed in inflamed human TMJ synovium tissue (right). Isotype-matched negative control antibodies were used under the same conditions. Scale bar = 100 μm.

TMJ synovial fluid and tissue samples were collected and analyzed from 19 patients who were undergoing TMJ surgery, arthroscopy or arthrocentesis (Table 1). We collected samples from 2 males and 17 females with ages ranging from 18 to 69 years-old and a mean age of 44 years-old. The majority or 80% of the patients were White, with 1 African American patient, 1 Hispanic patient, and 2 patients that reported race as unknown. TMJ pathology was reported based on clinical examination and imaging, including micro-CT or MRI. Patients were undergoing TMJ treatment for the following: osteoarthritis (n = 2), internal derangement and/or anterior disc displacement (n = 10), synovitis and/or capsulitis (n = 11), chronic dislocations (n = 1), synovial chondromatosis (n = 1), and chronic pain (n = 3). In one patient (H27L), arthrocentesis was performed and synovial fluid was extracted as a precaution due to the patient experiencing mandibular trauma, yet the patient had no prior history of TMJ disease and reported no underlying TMJ issues at the time of mandibular injury. The majority of the patients had multiple pathologies, whereby physical TMJ abnormalities like internal derangement and anterior disc displacement were often coupled with another diagnoses involving an inflammatory component, such as capsulitis and/or synovitis (n = 11). Interesting only 10% (2/20) of patients seeking TMJ treatment were diagnosed with TMJ pain, corroborating other findings suggesting that TMJ tissue pathology does not necessarily correlate with pain [23]. To confirm TMJ degeneration in the diseased human TMJ samples, we measured the release of the type II collagen C-terminal telopeptide and a biomarker for type II collagen degradation [24, 25] CTXII in the patients’ synovial fluid (Fig 1A). We classified TMJ conditions involving an inflammatory component in patients with either synovitis, osteoarthritis, and capsulitis; and we classified TMJ conditions without an active inflammatory component in patients as internal derangement, internal derangement, jaw contusion, and pain dysfunction syndrome (Table 1). Due to the difficulty of acquiring TMJ synovial fluid from a healthy human TMJ, we compared CTXII levels from patients presenting with inflammatory TMJ pathology (n = 12; inclusion criteria included synovitis, osteoarthritis, and capsulitis) to patients presenting with non-inflammatory TMJ pathology (n = 7; contusion, chronic dislocations, TMJ pain dysfunction syndrome, and disc displacement/internal derangement without associating synovitis). Relative to patients with pathology categorized as non-inflammatory, patients with pathology categorized as inflammatory showed elevated CTXII levels suggesting increased type II collagen degradation (Fig 1A) and plausible TMJ cartilage degeneration [26, 27]. Additionally, we measured the levels of the secreted inflammatory cytokine TNF-alpha in synovial fluid (Fig 1A) [28, 29]. In the 12 patients presenting with synovitis, capsulitis, or osteoarthritis (Table 1), there were elevated secreted TNF-alpha levels in synovial fluid relative to non-inflammatory controls. We further confirmed TNF-alpha expression in the synovial tissue derived from another patient with synovitis by immunohistochemistry and showed TNF-alpha expressed within the inflamed synovial tissue (Fig 1B). Taken together, these studies suggest increased cartilage degradation and high TNF-alpha levels are coupled and consistent among patients with TMJ pathologies that correspond to a subset of inflammatory tissue.

**Table 1:** Human TMJ synovial fluid.

Research ID	Age	Sex	Race	TMJ Pathology	CTX-II (pg/ml)	TNF-α (pg/ml)
H3	52	F	White	osteoarthritis (inflammtory)	97.5	31.2
H4	18	F	White	bilateral internal derangement with synovitis (inflammatory)	117.0	32.8
H4	30	F	White	anterior disc derangement and synovitis (inflammatory)	117.0	47.6
H6	69	F	White	osteoarthritis and capsulitis (inflammatory)	136.5	32.8
H7	42	F	Unknown	TMJ pain, capsulitis, internal derangement (inflammatory)	126.75	21.8
H8	32	F	White	synovitis, articular disc abnormality (inflammatory)	136.5	16.8
H9	23	F	White	chronic TMJ dislocations (non-inflammatory)	92.5	26.6
H10L	57	M	White	synovitis, anterior disc displacement (inflammatory)	136.5	15.6
H10R	57	M	White	synovitis, and anterior disc displacement (inflammatory)	156.0	0
H11	56	F	White	TMJ pain dysfunction syndrome (non-inflammatory)	97.5	0
H15	65	M	African American	synovial chondromatosis (inflammatory)	126.75	0
H16	66	F	White	internal derangement (non-inflammatory)	68.0	0
H17	66	F	White	internal derangement (non-inflammatory)	107.25	0
H19	30	F	White	TMJ synovitis; internal derangement (inflammatory)	117.0	15.6
H20	33	F	White	Anterior disc displacement without reduction, synovitis (inflammatory)	107.25	17.8
H22	26	F	White	Synovitis (inflammatory)	126.75	0
H23R	61	F	White	TMJ pain dysfunction syndrome (non-inflammatory)	117.0	7.8
H27L	37	F	White	Contusion of jaw (non-inflammatory)	73.0	7.8
H30	29	F	Hispanic	Anterior dis displacement (non-inflammatory)	97.5	0
H31	25	F	Unknown	TMJ synovitis, anterior disc displacement (Inflammatory)	78.0	0

### TNF-alpha induces extracellular matrix turnover and inflammatory cytokines in murine TMJ

**Fig 2:**
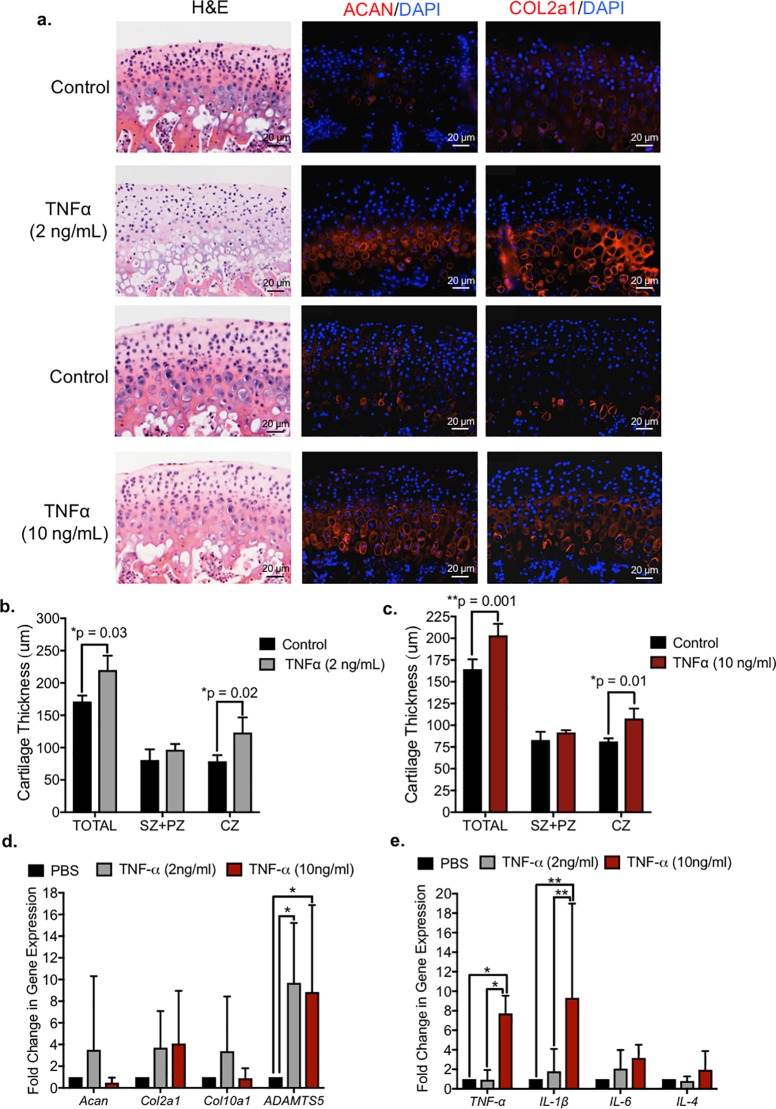
TNF-alpha increases extracellular matrix turnover and inflammatory cytokines in whole mandibular condyle explants. *(a)* Whole TMJ condyle explants were cultured for 48 hours in media supplemented with vehicle control, 2 ng/ml or 10 ng/ml rhTNF-alpha. Representative and comparable histological sections from explants were stained with H&E. Immunohistochemistry (red) showed increase in aggrecan (ACAN) and type II collagen (COL2a1) expression in explants treated with rhTNF-alpha. Scale bar = 20 μm. Isotype-matched negative control antibodies were used under the same conditions. *(b*,*c)* TMJ condyle explants treated with 2 ng/ml and 10 ng/ml *rh-TNF-alpha* had significantly increased total cartilage thickness and also within chondrocyte zone (CZ) as delineated by COL2a1+ expression relative to vehicle control explants. Data are mean ± SD; n = 3 explants; two-way ANOVA followed by Tukey’s post hoc. SZ = superficial zone; PZ = polymorphic zone; CZ = chondroctye zone as defined by Col2a1 expression. (*d*,*e)* qRT-PCR using total RNA extracted from TMJ condyle explants treated with vehicle, 2 ng/ml rhTNF-alpha and 10 ng/ml rhTNF-alpha. Data are normalized to GAPDH and mean fold change relative to vehicle ± SD; n = 4 explants; two-way ANOVA followed by Tukey’s post hoc. Explants treated with rhTNF-alpha showed significant increase in *ADAMTS5*, *TNF-alpha*, and *IL-beta* relative to vehicle controls.

To determine whether human TMJ pathology can be modeled in mice and define the contribution of TNF-alpha to TMJ pathology, we examined the effect of exogenous TNF-alpha on murine whole mandibular condyle explants (Fig 2). The mandibular condyle and approximately 3 mm of the neck of the ramus was dissected from the mandible and cultured in a 24-well plate overnight [8]. The culture media was supplemented with either 2 ng/mL or 10 ng/mL TNF-alpha for 48 hours, while vehicle PBS control was added to the contralateral explant (n = 7 explants). Immunohistochemistry showed that the expression of two extracellular matrix proteins (ECM) abundant in cartilage, aggrecan (ACAN) and type II collagen (COL2A1), increased in explants treated with either 2 and 10 ng/ml TNF-alpha relative to the contralateral vehicle control explants (Fig 2A). The total cartilage thickness significantly increased in explants treated with either 2 or 10ng/mL TNF-alpha, which was due to the significant expansion of the chondrocyte zone (CZ), marked by Col2a1 expression, but not due to expansion of the superficial and polymorphic zones (Fig 2B and 2C). Interestingly, the number of cells did not change in TNF-alpha treated explants (S1A and S1B Fig). These data suggest that an increase in cartilage thickness may be due to increased ECM deposition and chondrocyte differentiation, as opposed to cell proliferation. We speculated that TNF-alpha may also induce ECM turnover and inflammation in [30] TMJ condyle explants [8]. To corroborate this idea, we evaluated the gene transcripts of ECM constituents and inflammatory mediators of osteoarthritis by qRT-PCR. We found that *Adamts5*, *TNF-alpha*, and *IL-1beta* gene transcripts were significantly increased in explants treated with TNF-alpha relative to vehicle control explants (Fig 2D and 2E). These data suggest that TNF-alpha induces ECM turnover and inflammation in TMJ condyle explants.

**Fig 3:**
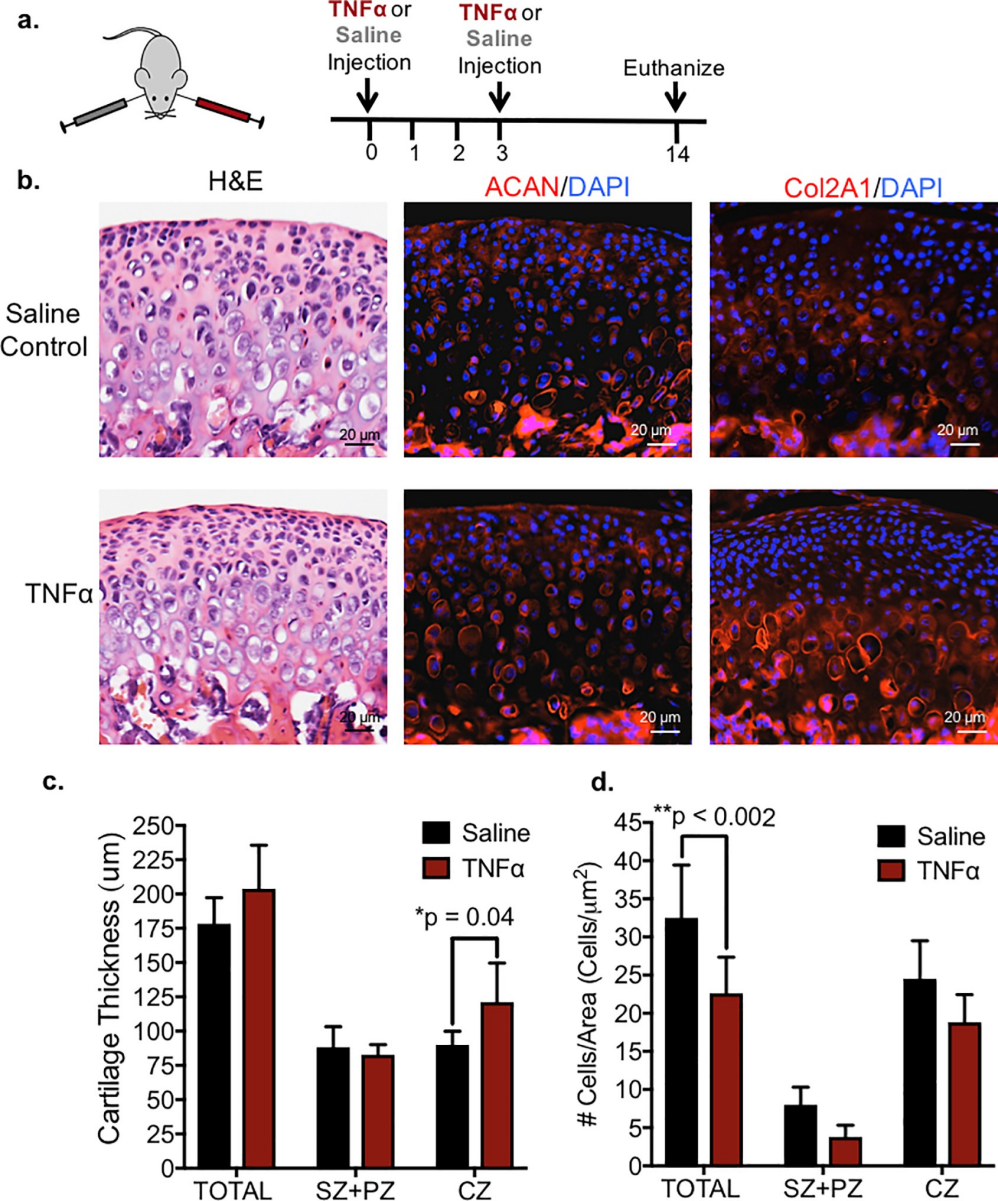
TMJ intra-articular injections of rhTNF-alpha promotes increased cartilage thickness and cell depletion in mandibular condyles. *(a)* rhTNF-alpha was injected intra-articularly into the TMJ unilaterally, while vehicle control was injected into the contralateral TMJ (n = 6 mice). *(b)* Representative and comparable histological sections from TMJs were stained with H&E. Immunohistochemistry (red) showed increase in aggrecan (ACAN) and type II collagen (COL2a1) expression in condyles treated with rhTNF-alpha. Isotype-matched negative control antibodies were used under the same conditions. *(c)* TMJs treated with rhTNF-alpha had significantly increased total cartilage thickness which was contributed to the expansion of the chondrocyte zone (CZ) as indicated by COL2a1+ expression relative to vehicle control explants. The superficial zone (SZ) and polymorphic zone (PZ) as indicated by COL2a1^-^ expression was not changed. Data are mean ± SD; n = 6 mice; two-way ANOVA followed by Tukey’s post hoc. *(d)* TMJs treated with rhTNF-alpha had significantly decreased number of cells within chondrocyte zone (CZ) and superficial and polymorphic zones (SZ+PZ) as indicated by COL2a1^+^ and COL2a1^-^ expression respectively relative to contralateral TMJs. Data are mean ± SD; n = 6 mice; two-way ANOVA followed by Tukey’s post hoc.

**Fig 4:**
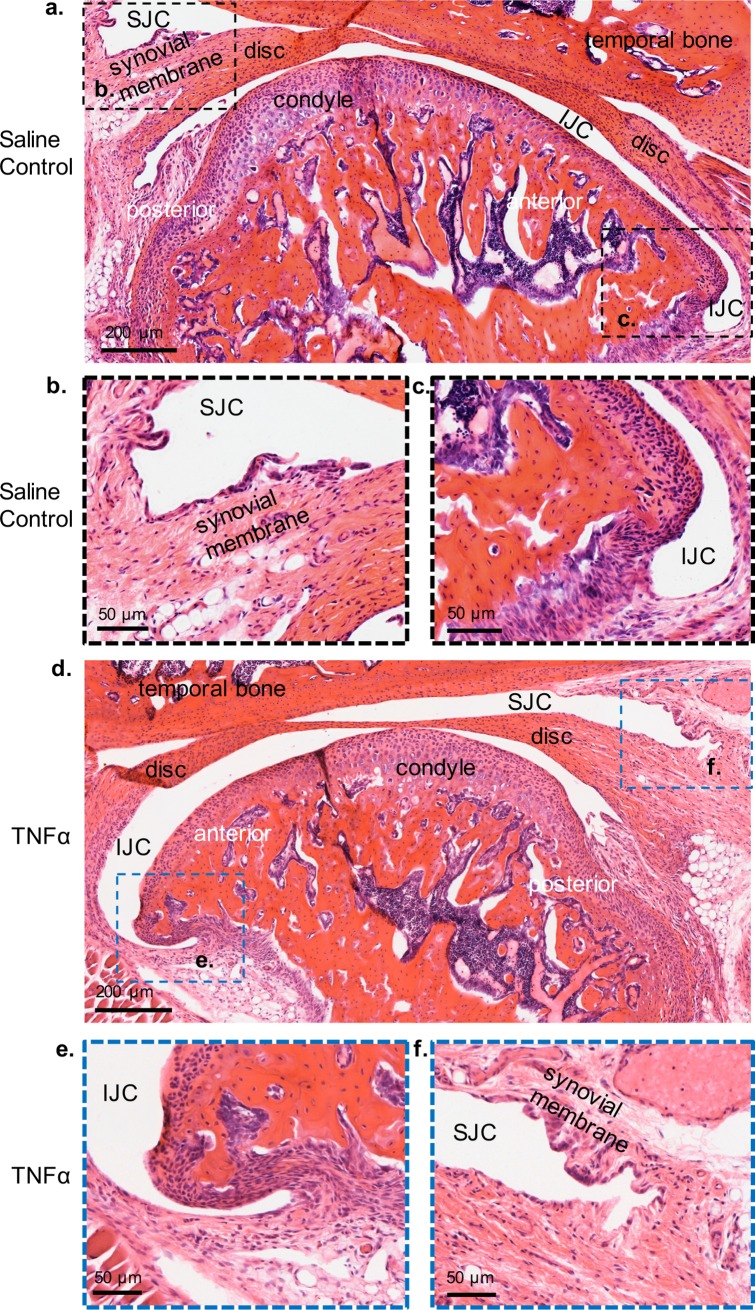
TMJ intra-articular injections of rhTNF-alpha induces minimal effects on surrounding TMJ tissues. H&E Sections from TMJs treated with saline control (*a-c*) and contralateral TMJ treated with rh-TNF-alpha (*d-f*). Black dashed box (*a*) is shown in higher magnification below (b,c). Blue dashed box (*d*) is shown in high magnification below (*e*,*f*). SJC = superior joint cavity; IJC = inferior joint cavity. No apparent changes in surrounding soft tissue were noted in TMJs treated with rhTNF-alpha relative to saline controls.

While whole condyle explants and organ cultures can provide insight into the chondrocyte proliferation and differentiation in response to the addition of exogenous cytokines and growth factors [8, 30–32], there are limitations to this approach in modeling joint pathology. drawbacks include the inability to culture long term, the absence of the contribution from joint soft tissues, and the inability to recapitulate cartilage matrix degradation and ossification in the terminal hypertrophic chondrocytes [30, 33, 34]. Therefore, to determine the effect of TNF-alpha *in vivo*, we injected TNF-alpha into the TMJ intra-articular space twice at 3 day intervals, while saline was injected on the contra-lateral side (S2 Fig and Fig 3A). The TMJ intra-articular injection site was anatomically located below the zygomatic arch and anterior to the ear canal (yellow circle, S2A and S2B Fig). The TMJ phenotype was analyzed 14 days following the initial injection. Similar to explants (Fig 2), immunohistochemistry showed that TMJs treated with TNF-alpha *in vivo* had increased aggrecan and type II collagen expression (Fig 3B) and a significant increase in the thickness of the chondrocyte zone (CZ) (Fig 3C) relative to saline controls. Unlike TNF-alpha explants (S1A and S1B Fig), TMJ intra-articular injections of TNF-alpha exhibited a significant reduction in cell number (Fig 3D) relative to contralateral TMJ controls. This difference may be due to the effects of TNF-alpha on the TMJ over an extended period of time, whereby explants were cultured for 2 days and the *in vivo* effects were tested over 14 days. Interestingly, the local application of TNF-alpha *in vivo* had no overt effects on surrounding TMJ tissues (Fig 4); compared to control, the treated condyle did not display thickening of the synovial membrane, infiltration of inflammatory cells, hard tissue changes such as pannus formation, or changes in the TMJ disc thickness (S3A and S3B Fig). Furthermore, differences in disc tissue thickness upon injections of TNF-alpha *in vivo* was not significant relative to saline controls (S3A Fig). Thus the local application of TNF-alpha to the TMJ in both explant model and *in vivo* showed similar effects on the TMJ.

### Complete Freund’s Adjuvant induces extracellular matrix deposition and soft tissue inflammation in murine TMJ

**Fig 5:**
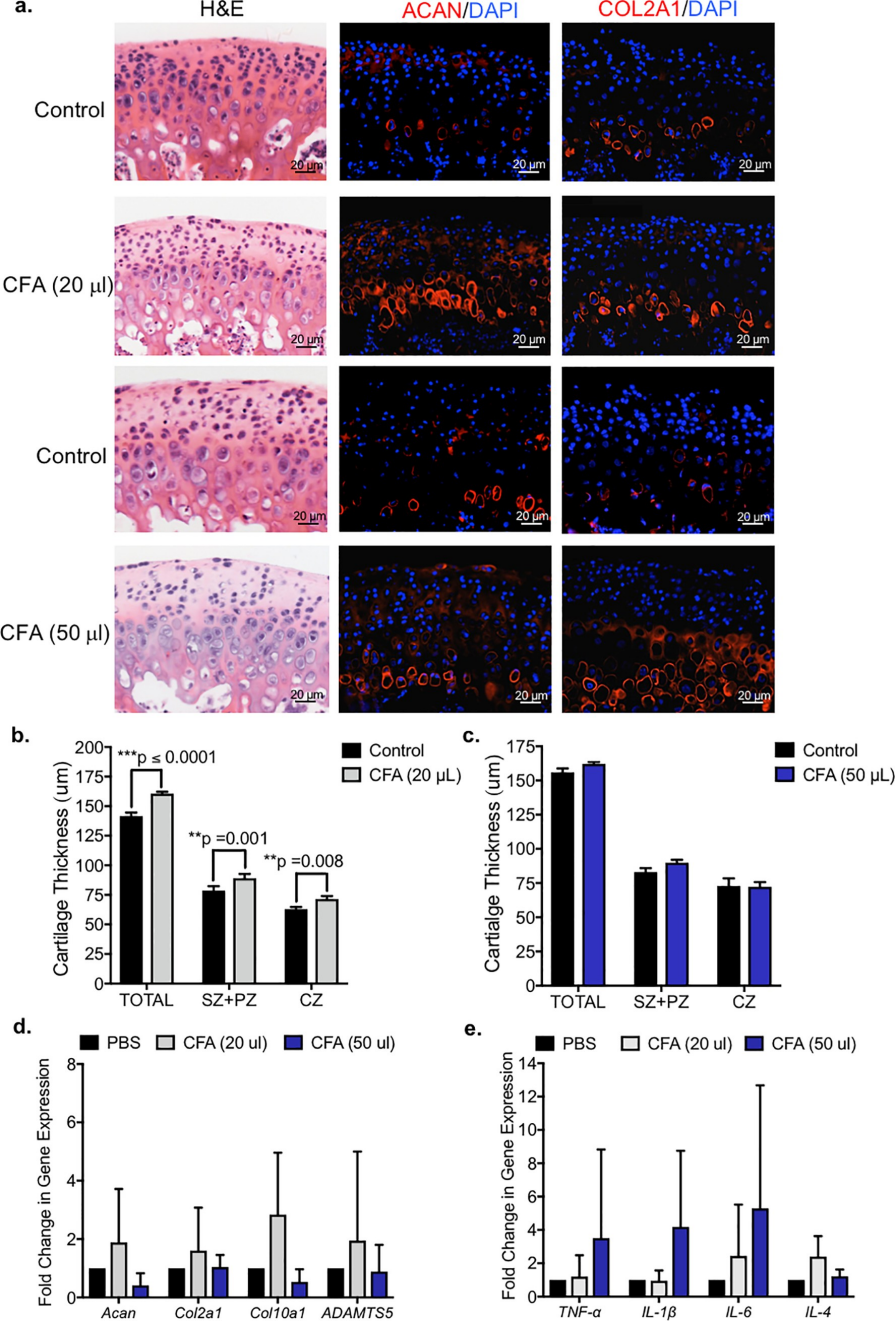
Complete Freund’s Adjuvant induces increased type II collagen expression in whole mandibular condyle explants. *(a)* Whole TMJ condyle explants were cultured for 48 hours in media supplemented with vehicle control, 20 μl or 50 μg/ml CFA. Representative and comparable histological sections from explants were stained with H&E. Immunohistochemistry (red) showed increase in aggrecan (ACAN) and type II collagen (COL2a1) expression in explants treated with CFA. Isotype-matched negative control antibodies were used under the same conditions. *(b*,*c)* TMJ condyle explants treated with 20 μl CFA had significantly increased cartilage thickness within chondrocyte zone (CZ), as indicated by COL2a1+ expression, and within the superficial zone and polymorphic zone (SZ+PZ), as indicated by COL2a1- expression, relative to vehicle control explants. The cartilage thickness was not changed in explants treated with 50 μl CFA. Data are mean ± SD; n = 3 explants; two-way ANOVA followed by Tukey’s post hoc. (*d*,*e)* qRT-PCR of TMJ condyle explants treated with vehicle, 20 μl or 50 μl CFA. Data are normalized to GAPDH and mean fold change relative to vehicle ± SD; n = 4 explants; two-way ANOVA followed by Tukey’s post hoc.

The local application of Complete Freund’s Adjuvant (CFA) to chemically induce arthritis and TMJ pathology is well-established [35, 36]. To determine the contribution of Complete Freund’s Adjuvant (CFA) to TMJ pathology, we examined the effect of exogenous CFA on murine mandibular condyle explants (Fig 5). The TMJ mandibular condyle and approximately 3 mm of the neck of the ramus was dissected from the mandible and cultured in a 24-well plate overnight [8]. The culture media was supplemented with either 20 or 50 μL CFA for 48 hours, while vehicle PBS control was added to the contralateral explant (n = 7 explants). Similar to TNFα treated explants, immunohistochemistry showed that aggrecan (ACAN) and type II collagen (COL2A1) expression were increased in explants treated with CFA relative to the contralateral vehicle control explants (Fig 5A). The total cartilage thickness significantly increased in explants treated with 20 μL CFA (Fig 5B), but cartilage thickness did not change in explants treated with 50 μl of CFA (Fig 5C) suggesting a dose-response. The increase in cartilage thickness in explants treated with 20 μL CFA was due to both the significant expansion of the superficial zone and polymorphic zone (SZ+PZ) and chondrocyte zones (CZ) (Fig 5B). Interestingly, similar to TNF-alpha treated explants, the number of cells did not change in CFA treated explants (S1C and S1D Fig), suggesting the cartilage expansion in explants treated with 20 μL CFA was not due to cell proliferation. To determine whether CFA induced ECM turnover and inflammation in TMJ condyle explants, we performed qRT-PCR to measure ECM-related gene transcripts (Fig 5D) and inflammation-related (Fig 5E) gene transcripts. qRT-PCR showed that there were no significant changes in ECM-related nor inflammation-related genes in CFA treated explants relative to vehicle control explants (Fig 5D and 5E). Taken together, our data suggested that CFA induced ECM deposition in mandibular explants, but did not induce changes in gene transcripts related to inflammation or ECM turnover.

**Fig 6:**
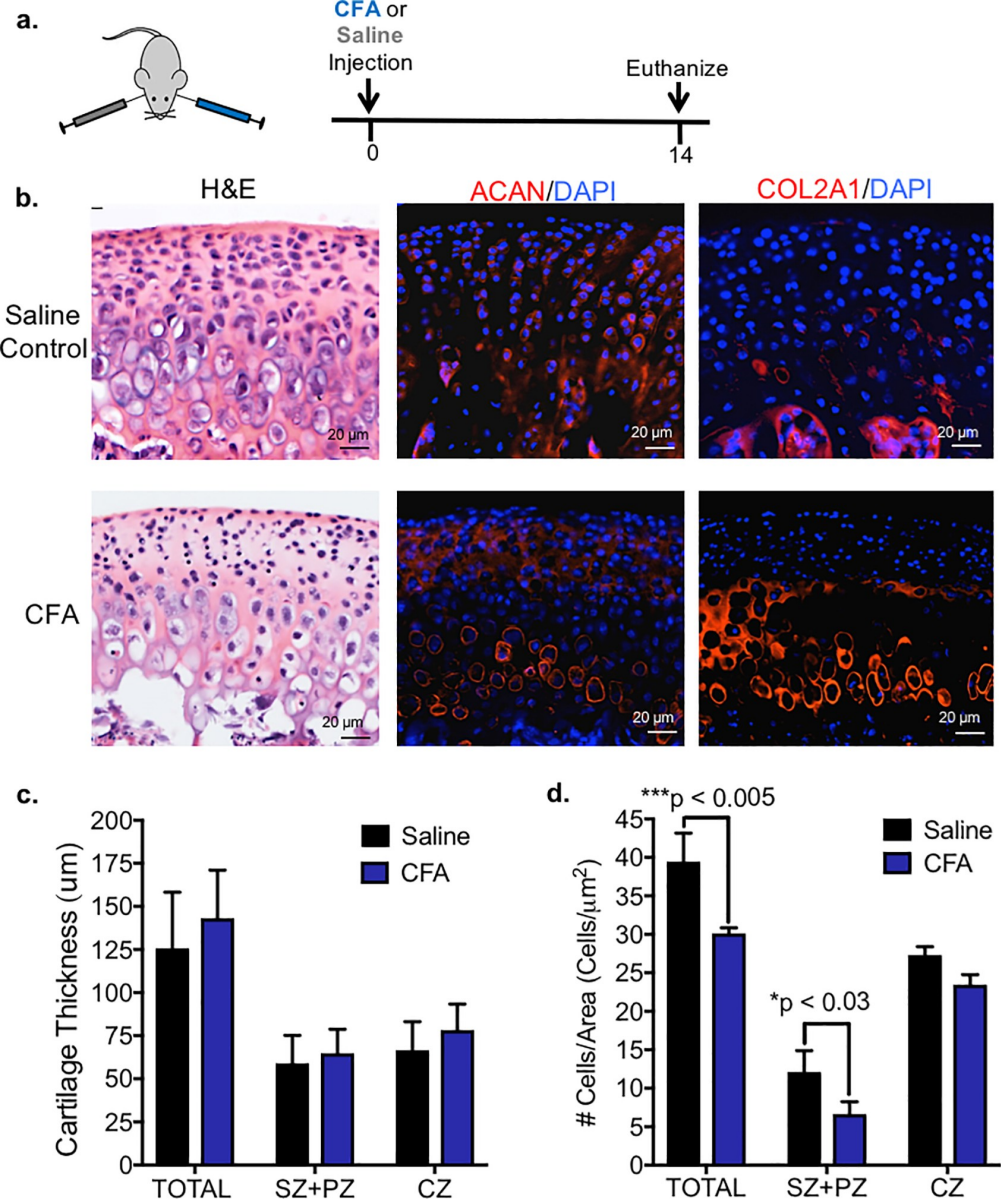
TMJ intra-articular injections of CFA promotes cell depletion in mandibular condyles. *(a)* CFA was injected intra-articularly into the TMJ unilaterally, while vehicle control was injected onto the contralateral TMJ (n = 4 mice). *(b)* Representative and comparable histological sections from TMJs were stained with H&E. Immunohistochemistry (red) showed increase in aggrecan (ACAN) and type II collagen (COL2a1) expression in condyles treated with CFA. Isotype-matched negative control antibodies were used under the same conditions. *(c)* TMJs treated with CFA had no change in cartilage thickness within all cellular zone (TOTAL), chondrocyte zone (CZ) as indicated by COL2a1+ expression, nor superficial zone and polymorphic zone (SZ+PZ) as indicated by COL2a1^-^ expression, relative to vehicle control explants. Data are mean ± SD; n = 4 mice; two-way ANOVA followed by Tukey’s post hoc. *(d)* TMJs treated with CFA had significantly decreased number of cells within total cartilage due to loss of cells in the superficial zone and polymorphic zones (SZ+PZ), as indicated by COL2a1^-^expression relative to contralateral TMJs. Data are mean ± SD; n = 4 mice; two-way ANOVA followed by Tukey’s post hoc.

**Fig 7:**
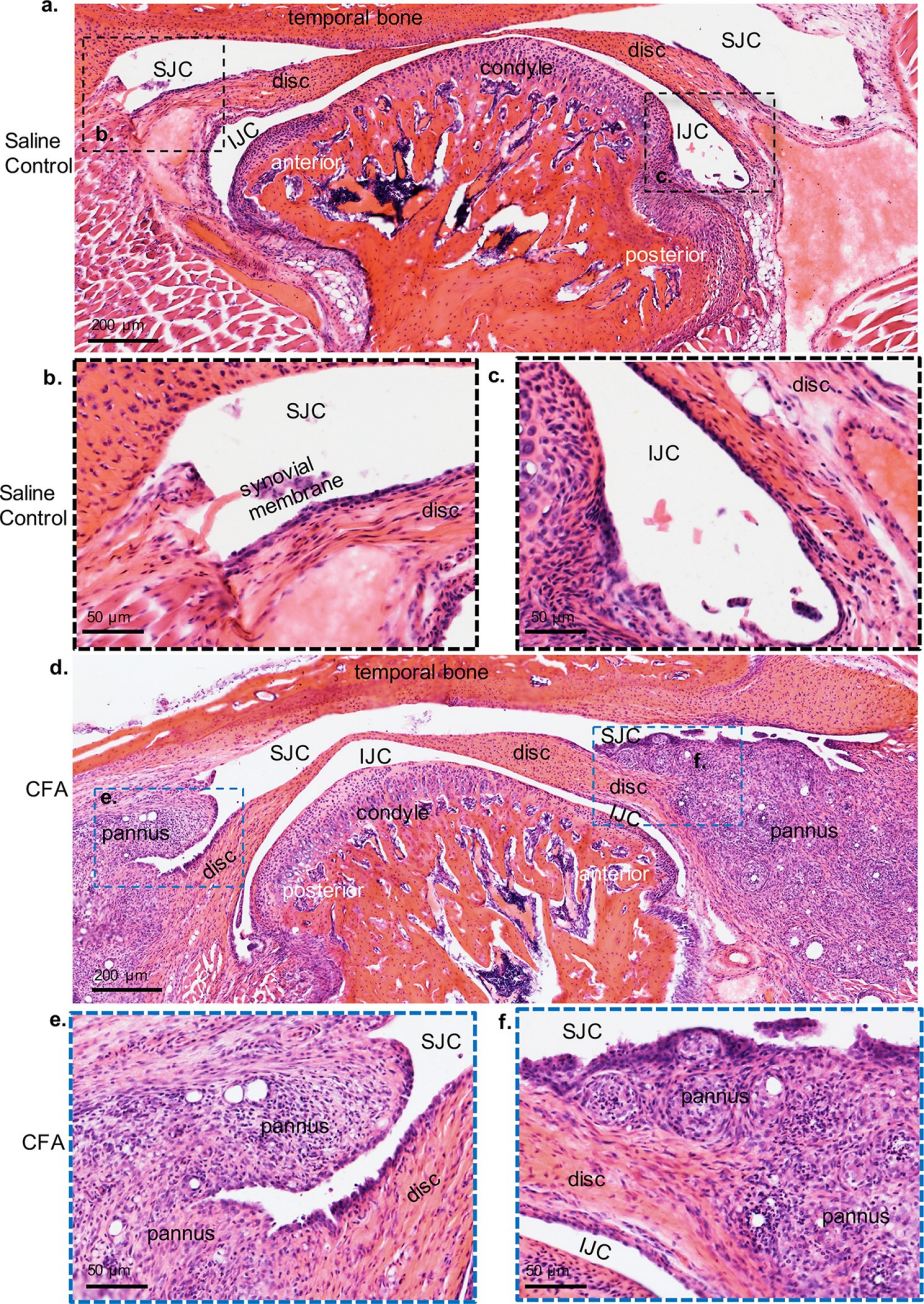
TMJ intra-articular injections of CFA promotes pannus formation on surrounding TMJ tissues. H&E Sections from TMJs treated with saline control (*a-c*) and contralateral TMJ treated with CFA (*d-f*). Black dashed box (*a*) is shown in higher magnification below (b,c). Blue dashed box (*d*) is shown in high magnification below (*e*,*f*). SJC = superior joint cavity; IJC = inferior joint cavity. TMJs treated with CFA showed arthritis phenotype, including pannus formation and synovitis within soft tissues surrounding TMJ.

To evaluate the effects of CFA to TMJ soft tissue and to substantiate the long term effects of CFA on the TMJ condyle, intra-articular injections of CFA were applied to murine TMJs *in vivo* (S2 Fig, Figs 2 and 6A). Similar to CFA treated explants, immunohistochemistry showed that the local injection of CFA to TMJs *in vivo* resulted in increased expression of aggrecan and type II collagen (Fig 6B). The cartilage thickness in CFA treated condyles was not changed relative to saline controls (Fig 6C), and there was a loss of cell number in the superficial and polymorphic zones (Fig 6D). Unlike TNF-alpha treated condyles (Fig 4), CFA treated condyles showed significant soft tissue changes, including pannus formation in both the anterior and posterior TMJ disc attachments and synovitis (Fig 7). Pannus tissue exhibited CD45+ cells relative to controls (S4 Fig) However, no difference was found in disc thickness in the anterior, intermediate, or posterior zones of the TMJ disc (S3A and S3B Fig). Thus, similar to other studies, we found local delivery of CFA into the TMJ induced soft tissue pathology [36].

## Discussion

Here, we show that a whole mandibular condyle explant model and an intra-articular injection model can be used to examine early, acute changes in inflammatory TMJ arthritis. In both models, we show that chemical induction of inflammation in the TMJ leads to extracellular matrix deposition and signs of cartilage degeneration. First, we show that the TMJ synovial fluid from diseased patients undergoing therapeutic arthroscopy or arthrocentesis has elevated levels of CTXII, a type II collagen degradative product that has been established as a marker for TMJ arthritis [37], and that TMJ synovial fluid from patients with inflammatory pathology has elevated levels of TNF-alpha. Given these findings, we speculated that direct delivery of TNF-alpha, which is involved in the pathogenesis of rheumatoid arthritis (RA) and known to stimulate fibroblast activity and fibrosis and a known chemical irritant, CFA, could replicate this phenotype in a mouse model [38] [39].

We show that in whole mandibular condyle explants, both ectopic TNF-alpha and CFA induce extracellular matrix deposition marked by increased type II collagen and aggrecan expression relative to control TMJs. Additionally, in the TNF-alpha delivery model, we observed an increase in the transcription of inflammatory cytokines. We also show that delivery of TNF-alpha and CFA via intra-articular injection into the TMJ space of mice induces acute changes in line with inflammatory TMJ arthritis. Local intra-articular injection of TNF-alpha and CFA induces extracellular matrix deposition, demonstrated by an expansion of type II collagen and aggrecan expression and a loss of cell density. Additionally, in the CFA intra-articular injection model, distinct soft tissue changes including inflammatory cell infiltrate, early pannus formation, and synovial membrane thickening are shown.

Chronic TMJ arthritis can lead to permanent loss of tissue over time. TMJ arthritis pathology is a dynamic process and involves abnormal ECM turnover, which results from an imbalance in ECM biosynthesis and degradation. Biosynthetic activities are often interpreted as the chondrocyte’s attempt to repair the damaged matrix [40, 41]. In the early stages of the disease, chondrocytes may undergo an increase in cellular proliferation [42] and an increase matrix synthesis [43–45]. Alternatively, chondrocyte activity can be destructive and may include increased cellular apoptosis [46] and the production of destructive cytokines like TNF-alpha [46–48] and interleukins [49, 50]. Additionally, upregulation of MMP activity, due to either an increase in MMP synthesis, increase activation of the proenzyme, or decrease inhibitor activity, may contribute to the cartilage ECM breakdown [51–53]. Ultimately, the biosynthetic chondrocyte activities are unable to sustain the functional demands of the tissue [54]. This results in a net loss of cartilage matrix components and the destruction and failure of the structural and functional properties of the cartilage. The present chemically induced TMJ arthritis mouse models do not display late stage degenerative disease, but rather early, acute changes including increased ECM deposition.

Our current study presents two models for studying chemically induced inflammatory TMJ arthritis in mice. We show that whole mandibular explants treated with varying concentrations of TNF-alpha and CFA display findings indicative of acute changes observed in TMJ arthritis. Whole mandible explant models offer significant advantages over primary chondrocyte monolayer cultures, given that explants maintain their native ECM structure and are phenotypically more stable [55]. Furthermore, another advantage of the *ex vivo* model is that it offers the unique similarities to the *in vivo* model, where the life-like evaluation of signals transmitted to and from the chondrocytes through the complex ECM tissue architecture. Chemically induced TMJ arthritis models have been reported in larger rat models [56] as opposed to mice, given the technical difficulty of TMJ intra-articular injections in smaller rodents. The only other reported models of adjuvant induced arthritis in mice utilize a method in which an adjuvant was delivered into the back-skin of mice or intraperitoneally, but not directly as an intra-articular injection [57, 58]. Here we develop the technique to inject into the mouse TMJ space with over 90% accuracy.

In addition to TNF-alpha and CFA, another chemical irritant commonly used to induce TMJ arthritis is mono-iodoacetate (MIA) [59]. However, MIA’s mechanism of action is inhibition of glyceraldehyde-3-phosphate dehydrogenase, which leads to apoptosis of chondrocytes. This mechanism of action causes a phenotype of destructive changes and cell death more similar to osteoarthritis or late-stage inflammatory arthritis than that observed in early stages of inflammatory arthritis, which we endeavored to explore in our model [60]. Therefore, our findings present a novel technique through which to examine an important disease process in a mouse model that can be further manipulated as needed for additional studies. However, our method is limited, as it does not display late changes noted in TMJ arthritis, such as bony changes and cartilage degradation, which are not present in our model.

When delivered via an intra-articular injection, both TNF-alpha and CFA provided similar phenotypes of acute TMJ inflammatory arthritis. However, one important distinction between treatment with TNF-alpha and CFA lies in the lack of obvious soft tissue pannus formation in the TNF-alpha treated mouse model. This suggests that CFA may provide more significant and sustained alterations in the inflammatory response compared to TNF-alpha. CFA is regarded as one of the best chemical irritants for the induction of chronic inflammation and is widely utilized [61]. However, a limitation in utilizing CFA for future studies lies in the unclear mechanism of how it produces inflammation. The bacterium in CFA, *Mycobacterium tuberculosis*, is believed to solicit a macrophage immune response that produces acute inflammation, but the immune response could also be induced by other components within the suspension [56]. The advantage of utilizing TNF-alpha to chemically induce inflammatory arthritis is that TNF-alpha is a major cytokine and signals a well-established signaling pathway [62–64]. Importantly, TNF-alpha is a crucial target in the medical management of patients with rheumatoid arthritis through medications like infliximab and etanercept [65]. Therefore, induction of inflammatory arthritis in a mouse model through delivery of TNF-alpha offers the potential to study the specific molecular underpinnings driving TMJ pathology. The lack of soft tissue changes and pannus formation in the TNF-alpha model could be addressed in future studies through increasing dosing frequency or increasing the delivered concentration of TNF-alpha. Modifications of the protocol through these avenues could achieve more dramatic soft tissue changes and sustained degenerative alterations.

One further limitation of our study is the utilization of the contralateral TMJ, injected with saline, as our control group. Studies suggest that CFA injection on one side can have a systemic effect on the contralateral condyle [66]. Additionally, any differences in loading or function due to the diseased condyle could be transmitted to the contralateral side. However, the design of the current study’s control prevents any potential differences being attributed to differences across experimental animals.

In summary, analysis of the present data is consistent with the hypothesis that delivery of chemical irritants to the TMJ condyle in mice using both a whole mandible explant model and local intra-articular injections can be utilized to study acute changes that mimic TMJ inflammatory arthritis. These methods replicate changes noted in the synovial fluid of human subjects representing inflammatory TMJ pathologies. Future studies that utilize the described models to further characterize the mechanisms underlying TMJ arthritis will enhance the current knowledge of TMJ disorders.

## Methods

### Human TMJ Samples

TMJ synovial fluid and tissue samples were collected and analyzed from 20 human patients who were undergoing either TMJ arthroscopy or arthrocentesis for TMJ therapy. After collection, samples were stored at -80°C until analysis could be completed. The study was approved by the Institutional Review Board of Columbia University Irving Medical Center (AAAQ8195) and the Institutional Review Board of Weill Cornell New York Presbyterian (1608017486-A002). All subjects were provided with written informed consent prior to study enrollment. All experiments were performed in accordance with relevant guidelines and regulations

### ELISA

The CTX-II (Aviva Systems Biology OKEH02711) and TNF-alpha (Boster EK0525) concentrations in the TMJ synovial fluid from each sample were measured using the quantitative sandwich enzyme immunoassay technique following manufacturer’s instructions. Briefly, standard or sample (100 μL) was added to each well and incubated for 2 h at 37°C, followed by treatment with primary antibody using either biotinylated anti-human TNF-alpha or 50 μL biotinylated anti-human CTXII antibody for 1 h at 37°C. For antibody detection, 100 μL of HRP-Avidin was for 1 h at 37°C. After washing, TMB substrate was added to each well for 15–30 min at 37°C, followed by the addition of 50 μL of Stop Solution. The optical density (OD) of each well was determined using a microplate reader at 450 nm. The CTX-II and TNF-alpha concentrations were calculated by comparing the OD of each sample to the standard curve.

### Animals

All animal procedures were performed with approval from IACUC at Columbia University Irving Medical Center (AC-AAAO4651; AC-AAAU6480). All experiments were performed in accordance with relevant guidelines and regulations. Wild type C57Bl/6 male and female mice were used for all experiments listed and were bred and genotyped according to recommended protocol (JAX #000664). Both males and female C57Bl/6 mice ages 6 weeks (n = 6), 8 weeks (n = 12), and 10 weeks (n = 4) were equally used for experiments. All animals were sacrificed with carbon dioxide gas followed by manual cervical dislocation after confirming that the animals were nonresponsive to alleviate potential suffering.

### Condyle explant model

TMJ condyles were harvested from male and female C57Bl/6 mice, age 6 weeks (n = 7) under sterile conditions. The articular condyle and approximately 3 mm of the neck of the ramus was dissected from the mandible and cultured in a 24-well plate using BGJb media (Thermo Fisher 12591038) supplemented with 1% penicillin/streptomycin (Invitrogen 15140–163). Condyle explants were incubated overnight (5% CO_2_, 37°C) to normalize to culture conditions. After overnight incubation, either 20 μL or 50 μL Complete Freund’s Adjuvant (CFA, Sigma F5881, 1:1 dilution) or 2 ng/mL or 10 ng/mL rhTNF-alpha (R&D 210-TA-020/CF) were added to one condyle explant with the contralateral condyle explant treated with the vehicle control (Henry Schein 1261699). Three condyles were treated with three freeze/thaw cycles for control. After 48 hours, condyle explants were either snap-frozen in liquid nitrogen for RNA isolation (n = 4 explants) or prepared for histology (n = 3 explants).

### Histology and immunohistochemistry

Tissue samples were fixed in 4% PFA in 1X PBS, decalcified in 0.5 M EDTA, embedded in OTC, and prepared for frozen sections. For histological analysis, samples were stained with H&E in compliance with the manufacturer’s protocol. H&E staining was performed to detect gross morphological changes, articular cartilage thickness, and synovitis. For immunohistochemistry, serial frozen sections were enzymatically treated with Chondroitinase ABCase (Fisher C3667-10UN) for 1 hour and immunolabeled with antibodies against Type II collagen (1:50, Millipore MAB8887), Aggrecan (1:100, Millipore AB1031), and CD45 (1:100, eBioscience^™^ 14-0451-81) in 10% goat serum at 4°C overnight followed by secondary antibody 37°C for 20 min (Invitrogen A-11010). Isotype-matched negative control antibodies were used under the same conditions. Vectashield mounting media with DAPI (4', 6-diamidino-2-phenylindole) was used for the nuclear counterstain.

### Histomorphometry

Using TMJ tissue sections immunolabeled with type II Collagen, the Col2a1^+^ chondrocyte zone (CZ) was delineated from the superficial and polymorphic zones (SZ+PZ) in order to determine if the distinct zones of the TMJ cartilage were differentially affected by treatment [20]. The thickness of the type II collagen positive chondrocyte zone (CZ) versus the type II Collagen negative superficial and polymorphic zones (SZ+PZ) was measured for the explant (n = 3 explant per group) and intra-articular injection model (n = 6 mice per TNF-alpha treatment group; n = 3 mice for CFA treatment group). Cell density (reported as # Cells/μm^2^) in various zones of the condyle was determined by manual counts of the DAPI positive nuclei within the Col2a1^+^ chondrocyte zone (CZ) versus the Col2a1^-^ superficial and proliferative zones (SZ+PZ) and using an area tool to select the delineated region and calculate the area (n = 3 explants/per experimental group; n = 6 mice for TNF-alpha treatment group; n = 3 mice for CFA treatment group). For TMJ disc measurements H&E sections were used and a blinded examiner measured disc thickness for the anterior, middle and posterior zone of the disc (n = 6 mice for TNF-alpha treatment group; n = 3 mice for CFA treatment group). For the anterior and posterior bands, the longest distance was measured. For the middle zone, the shortest distance was used for measurements. All measurements were quantified using Olympus cellSens Dimension imaging software and difference between groups was analyzed statistically using Prism 6 Graphpad Software as described below.

### RNA isolation and qRT-PCR

Total RNA was purified from TMJ condyle explants post-snap freezing in liquid nitrogen. Briefly, condyle explants (n = 4 mice per experimental group) were homogenized with Dounce homogenizers over dry ice and then combined with TriZol reagent (Thermo 15596026). RNA was then isolated from the homogenate using a standard protocol (Ambion 12183018A). RNA samples (260/280>1.8) were used to obtain cDNA (Biorad AM2222). Quantitative RT-PCR was performed using SYBR Green Master Mix (101414) and designed primers for *Acan*, *Col2a1*, *Col10a1*, *Adamts5*, *TNF-alpha*, *IL-1beta*, *IL-6*, and *IL-4* (S1 Table). Gene expression levels were normalized to housekeeping gene *Gapdh*.

### Inflammatory arthritis mouse models

Complete Freund’s Adjuvant (Sigma F5881) (CFA, 0.020 mL, 1:1 dilution, 10 weeks, n = 4 mice) or rhTNF-alpha (R&D 210-TA-020/CF) (0.020 mL, 0.5 μg/mL, 8 weeks, n = 6 mice) was injected unilaterally into the TMJ intra-articular space of C57Bl/6 mice once or twice respectively (S2 Fig). Saline was injected on the contralateral side as control. Animals were euthanized after 14 days and prepared for histology. Treated TMJs were processed for histology and examined by immunohistochemistry. To practice and validate the accuracy of our approach for murine TMJ intra-articular injections, 0.020 mL hematoxylin was injected into mouse cadaver TMJs (n = 24 mouse cadaver TMJs). Following injection, mouse cadaver TMJs were dissected to confirm and visualize hematoxylin within TMJ inferior joint space with over 90% accuracy (S2E and S2F Fig).

### Statistical analysis

All statistics were calculated using Prism 6 Graphpad Software. The statistical significance between two groups was determined using paired Student's t-test assuming Gaussian distribution. The normality of distribution was confirmed using Kolmogorov-Smirnov test and the resulting two-tailed P-value ≤ 0.05 was regarded as statistically significant difference. Among two groups, one-way ANOVA followed by Tukey’s post hoc test was used for statistical comparisons. For multiple comparisons, a two-way ANOVA followed by Tukey’s post hoc was used for statistical comparisons.

## Supporting information

S1 Table qRT-PCR primers. This is a table of all primers used in qRT-PCR experiments following the method discussed above.
(DOCX)

S1 Fig The total number of cells did not change in explants treated with either rhTNF-alpha or CFA. Whole TMJ condyle explants were cultured for 48 hours in media supplemented with vehicle control, 2 ng/ml or 10 ng/ml rhTNF-alpha (a,b) or 20 and 50 μL CFA (c,d). The total number of cells (TOTAL) within all cellular zones of maturation, superficial zone and polymorphic zone (SZ+PZ) and chondrocyte zone (CZ) are not change in explants treated with either 2 ng/ml or 10 ng/ml rhTNF-alpha (a,b) or 20 and 50 μL CFA (c,d), respectively. Data are normalized to GAPDH and mean fold change relative to vehicle ± SD; n = 3 explants; two-way ANOVA followed by Tukey’s post hoc.
(TIF)

S2 Fig Murine TMJ intra-articular injection technique. Complete Freund’s Adjuvant) (CFA, 0.020 mL, 1:1 dilution, 10 weeks, n = 4 mice) or rhTNF-alpha (R&D 210-TA-020/CF) (0.020 mL, 0.5 μg/mL, 8 weeks, n = 6 mice) was injected unilaterally into the TMJ intra-articular space of C57Bl/6 mice once or twice respectively. Saline was injected on the contralateral side as control. The TMJ intra-articular injection site was anatomically located below the zygomatic arch and anterior to the ear canal (yellow circle, a). Animals were euthanized after 14 days and prepared for histology. To validate the accuracy of our injection technique, we first confirmed the injection site by applying 20 μL of fast green into the intra-articular compartment (**b**). Dissection of the masseter and the portion of the zygomatic arch to reveal the TMJ showed fast green surrounded the TMJ capsule and intra-articular space (c,d). Dissection of the mandible showed fast green surrounded the TMJ condyle, validating our injection technique (e).
(PDF)

S3 Fig The TMJ disc thickness was unchanged upon intra-articular injections of rhTNF-alpha or CFA. rhTNF-alpha or CFA was injected intra-articularly into the TMJ unilaterally, while vehicle control was injected onto the contra-lateral TMJ (n = 4 mice TNF-alpha; n = 6 mice CFA). TMJ disc thickness was measure in the anterior, middle and posterior regions. There were no changes in TMJ disc thickness upon intra-articular injections of rhTNF-alpha (a) or CFA (b) relative to contralateral saline controls. Data are mean ± SD; n = 6 mice for rhTNF-alpha group and n = 4 mice for CFA group; two-way ANOVA followed by Tukey’s post hoc.
(TIF)

S4 Fig CD45 expression was upregulated in pannus formed upon TMJ intra-articular injection of CFA. CFA was injected intra-articularly into the TMJ unilaterally, while vehicle control was injected onto the contra-lateral TMJ (n = 6 mice). Immunohisochemistry showed CD45 was not expressed in saline treated TMJs (a). Immunohisochemistry showed CD45 was expressed and localized in pannus tissue formed in CFA treated TMJs (b).
(TIF)
