# Supplementary material for: Extracellular matrix turnover and inflammation in chemically-induced TMJ arthritis mouse models
Source: PLoS One. 2019 Oct 11;14(10):e0223244. doi: 10.1371/journal.pone.0223244 (PMC6788689; doi:10.1371/journal.pone.0223244)
Supplement: S1 Table — This is a table of all primers used in qRT-PCR experiments following the method discussed above. (DOCX) [file pone.0223244.s001.docx]

| **Supplemental Table 1: qRT-PCR Primers** | | |
| --- | --- | --- |
| Gene | FWD or REV | Sequence |
| *GAPDH* | Forward | GTGGAGATTGTTGCCATCAACGA |
| *GAPDH* | Reverse | CCCATTCTCGGCCTTGACTGT |
| *Col2a1* | Forward | GCAAGATGAGGGCTTCCATA |
| *Col2a1* | Reverse | CTACGGTGTCAGGGCCAG |
| *Col10a1* | Forward | ACCAGGAATGCCTTGTTCTC |
| *Col10a1* | Reverse | CATAAAGGGCCCACTTGCTA |
| *Adamts5* | Forward | GTCACATGAATGATGCCCAC |
| *Adamts5* | Reverse | CAAATGGCAGCACCAACATA |
| *Acan* | Forward | CCCTCAGAGTCACAAAGACCA |
| *Acan* | Reverse | TTCGCAGGGATAAAGGACTG |
| *IL-1beta* | Forward | GGTCAAAGGTTTGGAAGCAG |
| *IL-1beta* | Reverse | TGTGAAATGCCACCTTTTGA |
| *TNF-alpha* | Forward | AGGGTCTGGGCCATAGAACT |
| *TNF-alpha* | Reverse | CCACCACGCTCTTCTGTCTAC |
| *IL-6* | Forward | TGGTACTCCAGAAGACCAGAGG |
| *IL-6* | Reverse | AACGATGATGCACTTGCAGA |
| *IL-4* | Forward | CGAGCTCACTCTCTGTGGTG |
| *IL-4* | Reverse | TGAACGAGGTCACAGGAGAA |
