# Supplementary figures and images for: Extracellular matrix turnover and inflammation in chemically-induced TMJ arthritis mouse models
Source: PLoS One. 2019 Oct 11;14(10):e0223244. doi: 10.1371/journal.pone.0223244 (PMC6788689; doi:10.1371/journal.pone.0223244)

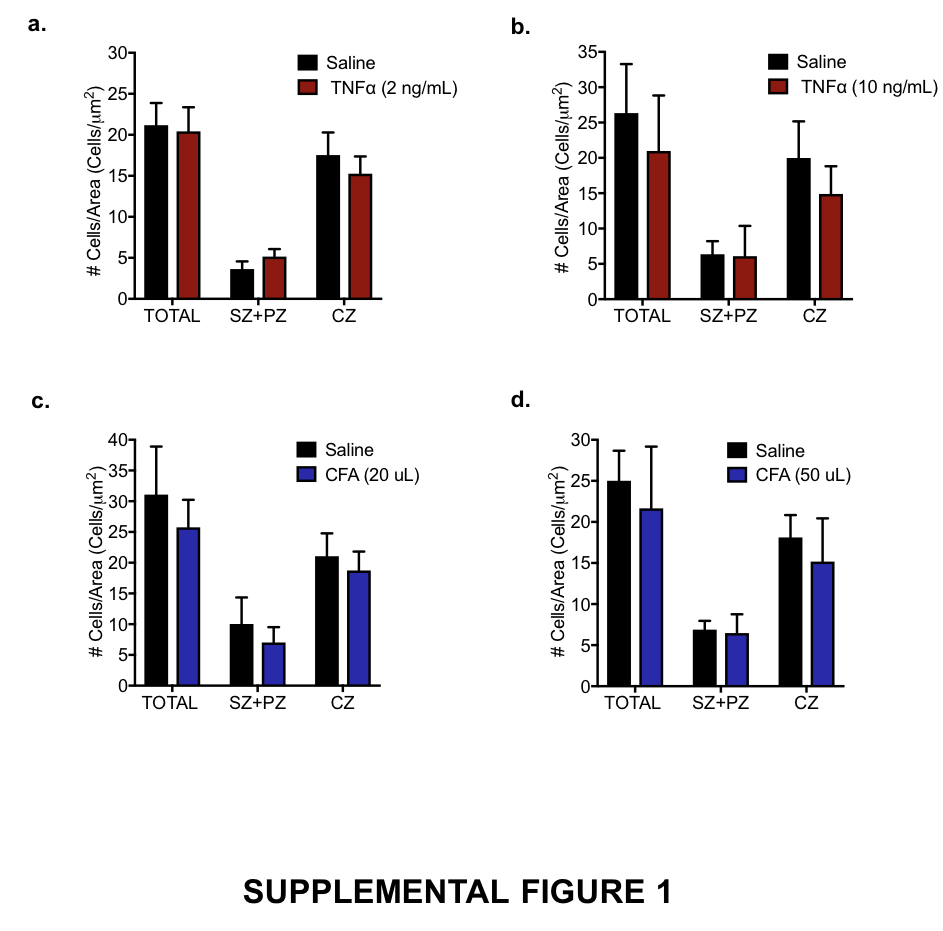

Supplement: S1 Fig — Whole TMJ condyle explants were cultured for 48 hours in media supplemented with vehicle control, 2 ng/ml or 10 ng/ml rhTNF-alpha (a,b) or 20 and 50 μL CFA (c,d). The total number of cells (TOTAL) within all cellular zones of maturation, superficial zone and polymorphic zone (SZ+PZ) and chondrocyte zone (CZ) are not change in explants treated with either 2 ng/ml or 10 ng/ml rhTNF-alpha (a,b) or 20 and 50 μL CFA (c,d), respectively. Data are normalized to GAPDH and mean fold change relative to vehicle ± SD; n = 3 explants; two-way ANOVA followed by Tukey’s post hoc. (TIF) [file pone.0223244.s002.tif]

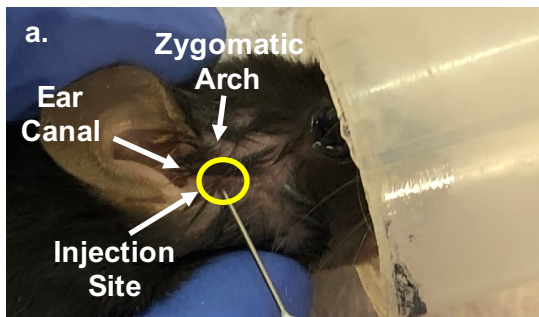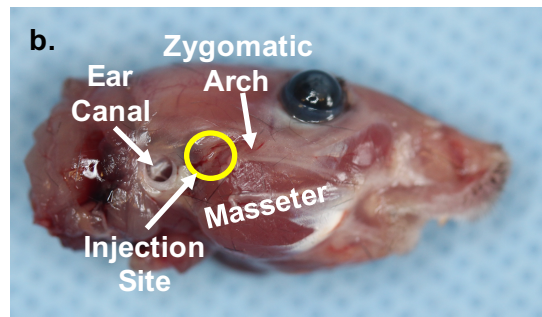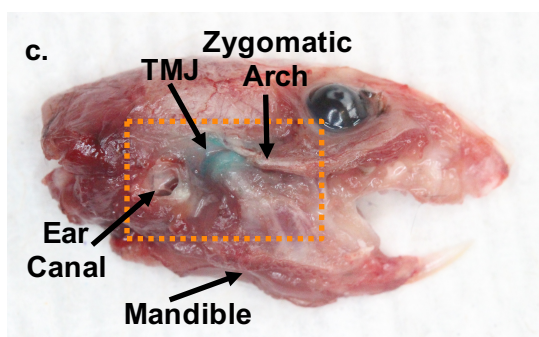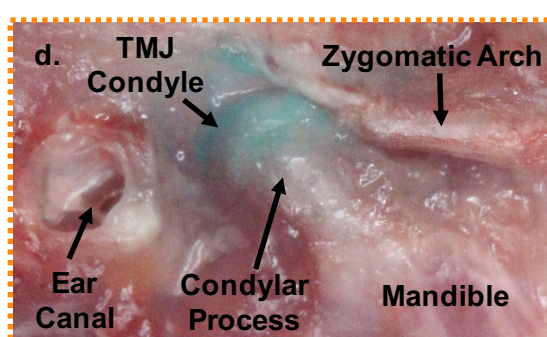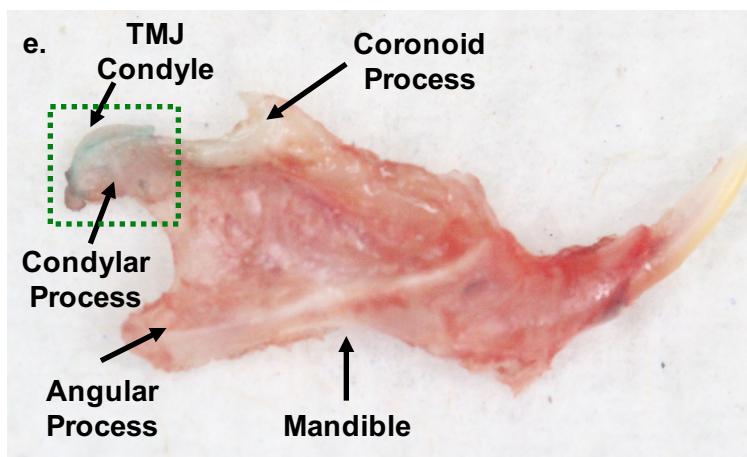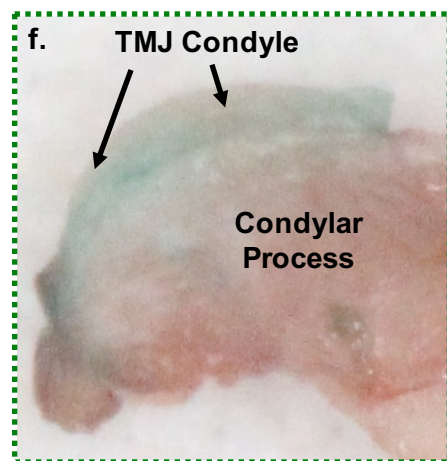

**SUPPLEMENTAL FIGURE 2**

Supplement: S2 Fig — Complete Freund’s Adjuvant) (CFA, 0.020 mL, 1:1 dilution, 10 weeks, n = 4 mice) or rhTNF-alpha (R&D 210-TA-020/CF) (0.020 mL, 0.5 μg/mL, 8 weeks, n = 6 mice) was injected unilaterally into the TMJ intra-articular space of C57Bl/6 mice once or twice respectively. Saline was injected on the contralateral side as control. The TMJ intra-articular injection site was anatomically located below the zygomatic arch and anterior to the ear canal (yellow circle, a). Animals were euthanized after 14 days and prepared for histology. To validate the accuracy of our injection technique, we first confirmed the injection site by applying 20 μL of fast green into the intra-articular compartment (b). Dissection of the masseter and the portion of the zygomatic arch to reveal the TMJ showed fast green surrounded the TMJ capsule and intra-articular space (c,d). Dissection of the mandible showed fast green surrounded the TMJ condyle, validating our injection technique (e). (PDF) [file pone.0223244.s003.pdf]

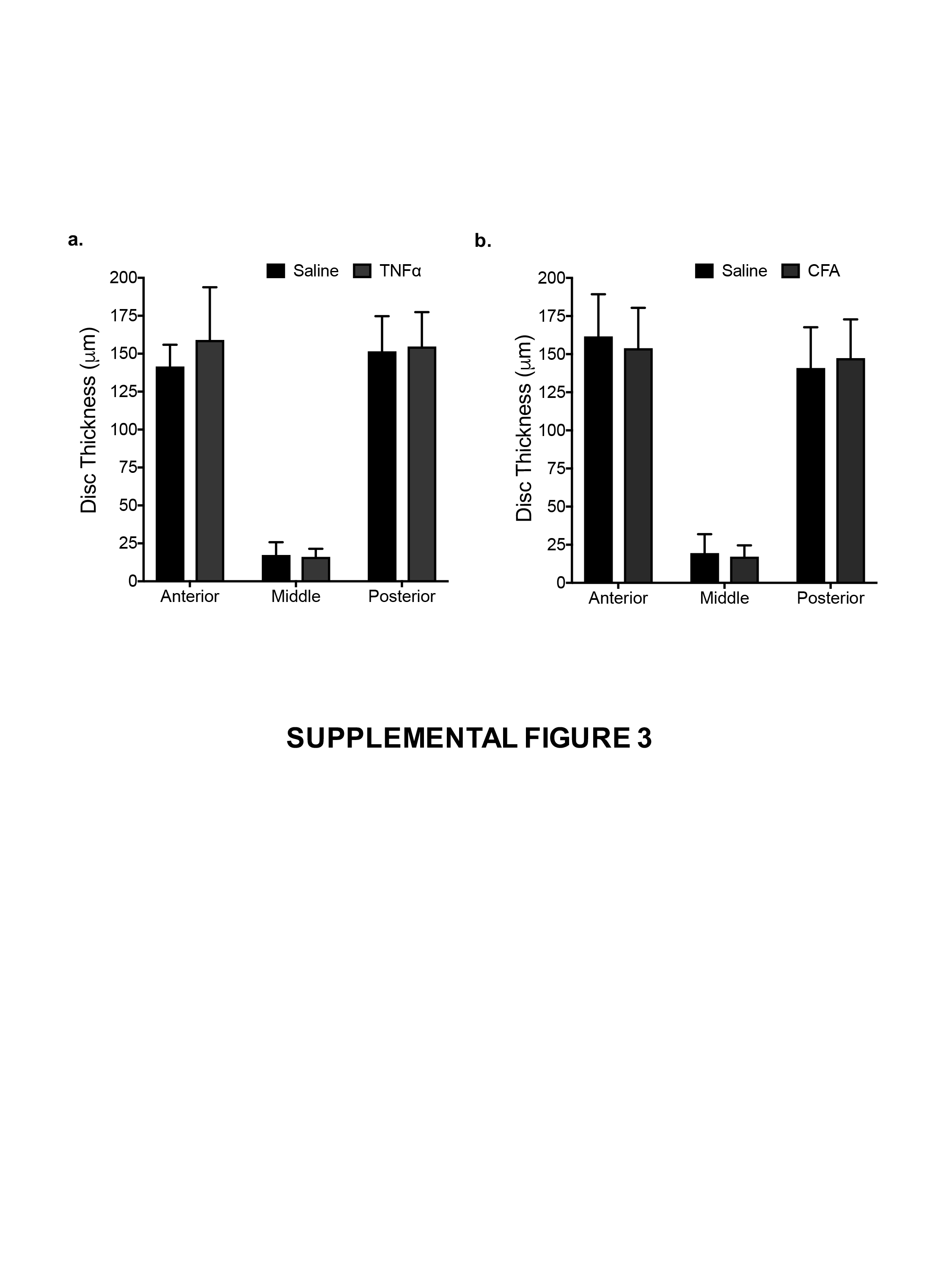

Supplement: S3 Fig — rhTNF-alpha or CFA was injected intra-articularly into the TMJ unilaterally, while vehicle control was injected onto the contra-lateral TMJ (n = 4 mice TNF-alpha; n = 6 mice CFA). TMJ disc thickness was measure in the anterior, middle and posterior regions. There were no changes in TMJ disc thickness upon intra-articular injections of rhTNF-alpha (a) or CFA (b) relative to contralateral saline controls. Data are mean ± SD; n = 6 mice for rhTNF-alpha group and n = 4 mice for CFA group; two-way ANOVA followed by Tukey’s post hoc. (TIF) [file pone.0223244.s004.tif]

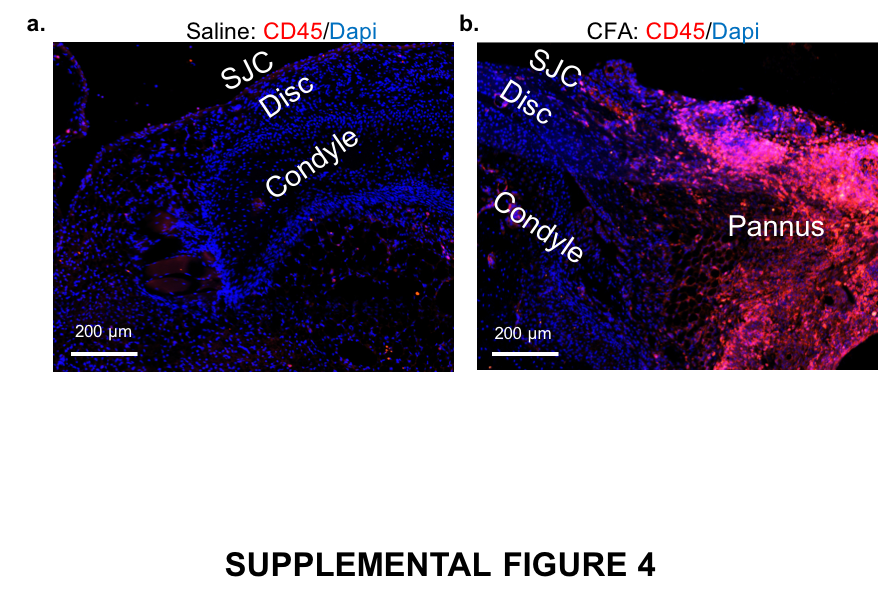

Supplement: S4 Fig — CFA was injected intra-articularly into the TMJ unilaterally, while vehicle control was injected onto the contra-lateral TMJ (n = 6 mice). Immunohisochemistry showed CD45 was not expressed in saline treated TMJs (a). Immunohisochemistry showed CD45 was expressed and localized in pannus tissue formed in CFA treated TMJs (b). (TIF) [file pone.0223244.s005.tif]
